# Chronic post-operative iris prosthesis endophthalmitis in a patient with traumatic aniridia: a case report

**DOI:** 10.1186/s12886-016-0383-1

**Published:** 2016-11-09

**Authors:** Kevin C. Firl, Sandra R. Montezuma

**Affiliations:** Department of Ophthalmology and Visual Neurosciences, University of Minnesota, 420 Delaware Street SE, MMC 493, Minneapolis, MN 55455 USA

**Keywords:** Iris prosthesis, Chronic post-operative endophthalmitis, Propionibacterium acnes, Case report

## Abstract

**Background:**

Post-operative endophthalmitis is a serious complication of intraocular surgery which may present acutely or chronically. Chronic post-operative endophthalmitis is characterized by decreased visual acuity, mild pain, and low-grade uveitis several weeks or months after intraocular surgery which may be responsive to corticosteroids, but recur upon tapering. Low virulence organisms such as *Propionibacterium acnes* are the most common culprit organisms, and treatment most often consists of both intravitreal antibiotic injections and surgery.

Aniridia is a condition defined by total or partial loss of the iris and leads to decreased visual quality marked by glare and photophobia. Treatment of complex or severe cases of traumatic aniridia in which surgical repair is difficult may consist of implantation of iris prostheses, devices designed to reduce symptoms of aniridia.

Though chronic, post-operative endophthalmitis has been associated with most intraocular surgeries including intraocular lens implantation after cataract removal, it has never been described in a patient with an iris prosthesis.

**Case Presentation:**

In this case report, we describe the case of a 49 year old, male construction worker with traumatic aniridia who experienced chronic, recurrent low-grade intraocular inflammation and irritation for months after implantation of the Ophtec 311 prosthetic iris. Symptoms and signs of inflammation improved temporarily with sub-Tenon’s capsule triamcinolone injections. Ultimately after more than 2 post-operative years, the iris prosthesis was explanted, and intravitreal cultures showed *P. acnes* growth after 5 days. Intravitreal antibiotics treated the infection successfully.

**Conclusions:**

To our knowledge, this is the first reported case of chronic, post-operative endophthalmitis in a patient with an iris prosthesis. Chronic, post-operative endophthalmitis may be a difficult to identify in the context of traumatic aniridia and iris prosthesis implantation due to other potential etiologies of chronic intraocular inflammation such as implant-induced chafing. Clinicians should suspect chronic, post-operative endophthalmitis in any case of recurrent, low-grade intraocular inflammation.

## Background

Post-operative endophthalmitis is a serious complication of intraocular surgery in which the vitreous cavity becomes infected and inflamed. Most of the time it presents acutely, within 90 post-operative days, with visual loss, pain, and hypopyon, but it may also occur in a chronic form, several weeks or months after surgery [1]. The incidence for acute, post-operative endophthalmitis (APOE) varies according to procedure, location, and time, and has been reported to be between 0.029 % and 0.22 % for cataract surgery [1–4], 0.046 % or 0.052 % for pars-plana vitrectomy [5, 6], and an average of 0.093 % for all intraocular procedures [6]. The incidence of chronic post-operative endophthalmitis (CPOE), though known to comprise a small portion of endophthalmitis cases overall, is unknown [7–9].

CPOE is characterized by decreased visual acuity, mild pain, and a recurrent low-grade uveitis often without hypopyon several weeks or months after surgery [10]. Keratic precipitates, conjunctival injection, and vitreous inflammation are also common findings [11]. White intracapsular plaques or pericapsular “pearls-on-a-string” findings may also be seen in cases of bacterial or fungal etiology, respectively, although neither finding is pathognomonic [8, 9]. Although these inflammatory findings may be somewhat responsive to corticosteroids, they are very likely to recur as soon as tapering begins.

Infectious agents associated with CPOE are generally slow-growing, non-invasive bacteria or fungi such as *Propionibacterium* species, *S. epidermidis*, *Candida parapsilosis*, and *Corynebacterium* species with *P. acnes* reported as the most common in four studies [10, 12–14]. If CPOE is suspected, aqueous and vitreous samples should be taken to identify culprit organisms and their antimicrobial sensitivities. In addition to Gram stain, samples should be sent for aerobic and anaerobic bacterial and fungal cultures and be monitored for a minimum of 2 weeks since many organisms including *P. acnes* may take more than a week to show culture positivity [7, 11].

Treatment for patients with CPOE is often medical and surgical. Conservative treatment consists of intravitreal antibiotics (IVAB) only, usually vancomycin for bacterial organisms or amphotericin B for fungal organisms. However, average recurrence rates after IVAB were 90 % according to one review [8]. In the same analysis, addition of pars plana vitrectomy, partial or total capsulectomy, and intra-ocular lens removal decreased recurrence rates dramatically, as low as 4.5 % if all interventions were performed. Visual acuity outcomes are generally better in eyes with CPOE than in those with APOE [7, 8]. In one study with 118 patients, post-treatment acuity of 20/40 or better was present in 50 % of CPOE patients and only 27 % of APOE patients. However, 89 % of those with APOE had a presenting visual acuity of 5/200 or worse, a far greater percentage than the 31 % of those with CPOE with 5/200 or worse visual acuity [13].

Aniridia is a condition in which portions of the iris or its entirety are absent. Without a fully functioning iris to act as a filter in bright conditions, patients with aniridia often experience decreased visual acuity, contrast sensitivity, and depth of focus and increased glare and photophobia. Aniridia may be congenital, but is most often acquired through severe ocular trauma. Although some iris defects, often smaller, simple injuries, may be repaired by surgery, large or complex traumatic defects may benefit from iris prostheses. Iris prosthetics are implants designed to look like an iris and improve symptoms of aniridia by blocking excess, incoming light. They may be implanted within the anterior chamber, capsule, or ciliary sulcus and sutured to the sclera or remnant iris [15, 16]. Current designs either include an intraocular lens or may be attached to one since few cases of traumatic aniridia spare the natural lens [17]. Currently, none of the handful of iris prosthetics available for use in Europe are approved by the FDA, so use within the US is limited to clinical trial participants or those for whom a compassionate use waiver is filed [16].

In this report, we present a patient who received an iris prosthesis implant after sustaining severe intraocular injury and experienced recurrent intraocular inflammation over the course of more than two years before undergoing explantation which revealed *p. acnes* infection.

## Case Presentation

**Fig. 1:**
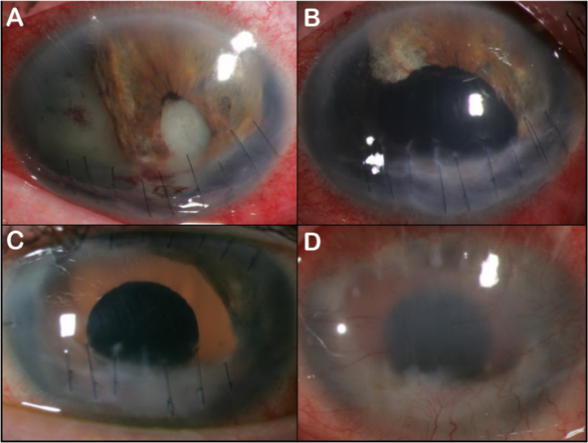
Slit lamp photography of left-eye **a** post-traumatic globe repair; **b** post- pars plana vitrectomy, iridiocorneal adhesion lysis, and lensectomy; **c** post- Ophtec 311 iris prosthesis implant; and **d** 2 years after implantation of iris prosthesis notable for vascularization of cornea and retrocorneal fibrous membrane

A 49 year old, male construction worker with no prior intraocular surgeries first presented to the emergency department after accidentally being struck in the left eye with a utility knife while at work. Upon examination, visual acuity (VA) of the right eye and left eye (OS) were 20/30 and hand movements (HM), respectively. Slit lamp examination showed a penetrating corneal laceration inferiorly with prolapsed iris and 270 degree iridodialysis. The patient was taken to the operating room for surgical repair of the open globe. Post-surgically, the patient was noted to have traumatic aniridia with remnants of iris superiorly and nasally only (see Fig. 1a). At follow-up appointments, the patient complained of occasional mild pain in the OS, but there were no signs of acute infection.

Three months after initial injury, a pars plana vitrectomy, lysis of iridocorneal adhesions, and lensectomy were performed due to chronic vitreous hemorrhage, post-surgical or inflammatory adhesions, and a traumatic cataract, respectively. The posterior capsule was removed and the anterior capsule opened. There were no acute complications (see Fig. 1b). At a two-week post-surgical follow-up appointment, the patient’s OS had a VA of 20/200 and an intraocular pressure of 10 mmHg. Slit lamp examination findings including diffuse conjunctival injection, mild corneal edema, and a deep anterior chamber were noted.

Because of the patient’s light sensitivity, aniridia, and aphakia, it was decided to implant an iris prosthesis with lens, the Ophtec 311. Implantation surgery took place two months after the prior surgery, five months since initial injury. For implantation, the Ophtec 311 was sutured into two scleral flaps made nasally and temporally 180 degrees away from each other and then centered onto the remaining anterior lens capsule and ciliary sulcus. The procedure was without acute complications and the patient was discharged with a prescription of dexamethasone drops every 2 hours and ofloxacin four times daily (see Fig. 1c).

On post-operative day 1 the VA was HM, IOP 7 mmHg, and there was 4 + cells and flare in the anterior chamber. These findings were unchanged after 2 weeks. At post-operative week 5, VA improved to 4/200, pain and photophobia were said to be improving, but 1+ cell and 2+ flare were noted. At post-operative week 6 and 8, the OS had a VA of 4/200, 6 mmHg IOP, and 2+ flare in the anterior chamber. Conjunctival injection and a hazy view through the Ophtec 311 lens were noted. The patient was offered a sub-Tenon’s capsule injection of triamcinolone, but declined.

By post-implant month 4, a retrocorneal fibrous membrane and 1+ corneal edema was seen in addition to persistent 1+ cell, 2+ flare, and a decline of VA to 1/200. Consequently, dexamethasone drops were increased from 3 to 4 times daily. At post-implant month 6, the patient complained of increasing frequency of pain and tearing, and examination findings included a VA of HM, 4 mmHg IOP, persistent corneal edema, and anterior chamber inflammation. A Sub-Tenon’s triamcinolone injection was performed for what was thought to be chronic uveitis secondary to implantation and trauma. By 3 months post-injection (9 months post-operative) the patient felt well, and the anterior chamber was clear on examination. Consequently, a slow taper of the dexamethasone drops was begun.

At post-implant month 14, the patient came to the emergency department because of a week of worsening redness, pain, and photophobia in the OS. On examination there was marked episcleral and conjunctival injection, diffuse corneal edema with deep and superficial vascularization, and as previously noted, a retrocorneal fibrous membrane and stable fibrous deposits on the Ophtec lens. There was a hazy view of the anterior chamber to assess for cells. The patient was given another sub-Tenon’s capsule triamcinolone injection, restarted dexamethasone drops four times daily, and started erythromycin ointment four times daily. Symptoms began to improve within a week, but the patient endorsed intermittent pain beginning a couple months later.

At post-implant month 21, the patient presented again in the emergency department because of a new foreign body sensation on the surface of his OS in addition to several weeks of intermittent pain and irritation of his OS. A slightly protruding suture from the iris prosthesis was found and trimmed. Consequently the foreign body sensation and pain improved, but intermittent pain, irritation, and mild conjunctival injection of the left eye continued through post-implant month 24 (see Fig. 1d). The patient was given another sub-Tenon’s capsule triamcinolone injection and explantation surgery was planned because of the patient’s chronic, intraocular inflammation.

At post-implant month 26, a penetrating keratoplasty, pars plana vitrectomy, and Ophtec 311 explantation surgery was performed. A penetrating keratoplasty was also performed due to the progressively worsening corneal edema and growth of a retrocorneal membrane. A Gram stain, aerobic, anaerobic, and fungal cultures were taken from vitreous. Aerobic, anaerobic, and fungal cultures of the cornea were also ordered. The patient tolerated the procedure well and there were no acute complications.

On day 5 of intravitreal culture, Gram positive bacilli were seen on the anaerobic vitreous culture medium and identified as *Propionibacterium acnes*. No organisms and rare white blood cells were seen on vitreous gram stain, and no growth was seen on cornea cultures. Pathologic examination of the cornea showed moderate endothelial cell loss, bullous keratopathy, inflammatory pannus, with stromal scarring and neovascularization.

The patient was treated with a 10 day course of levofloxacin, 750 mg daily, and intravitreal injections of 4.5 mg of ceftazidime and 2 mg of vancomycin. Two weeks after surgery, VA was stable at HM, there was 2+ flare and cells, and the patient was experiencing mild pain in the OS. By post-operative week 4, there were rare cells in the anterior chamber on examination and only occasional mild irritation reported by the patient.

**Fig. 2:**
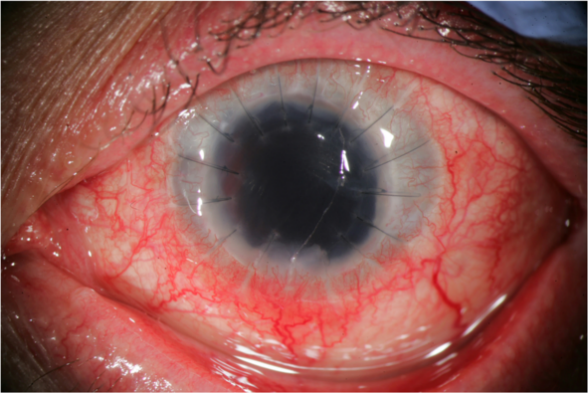
Slit lamp photography of left eye post explantation and keratoplasty

Most recently, at post-operative month 10, the corneal transplant was noted to have become edematous and failed, most likely due to keratoplasty performed in the setting of CPOE, so a good examination of the anterior chamber to completely rule out inflammation was not possible. However, the eye was quiet with no signs of recurrent inflammation, ultrasound showed an attached retina with no vitreous debris, and the patient reported complete resolution of ocular pain (Fig. 2).

## Conclusions

We have presented a patient who, after sustaining traumatic aniridia, receiving an Ophtec 311 iris prosthesis, and experiencing 2 years of recurrent intraocular inflammation and pain, was discovered to have culture-positive CPOE and treated successfully with removal of the Ophtec 311 and intravitreal antiobiotics. Although CPOE has been described in association with a variety of intraocular procedures as described above, to our knowledge this is the first time it has been reported in connection with a post-traumatic, iris prosthesis implantation leading to explantation.

In a phase I clinical trial for the Ophtec 311 iris prosthesis implanted in 10 eyes, there were no reported cases of endophthalmitis after a year of follow-up, but there were 2 cases of iritis, one at post-operative day 1 and the other at post-operative month 6, which resolved with topical corticosteroid treatment [18]. In studies following a total of nearly 158 patients implanted with a different iris prosthesis (the Morcher iris diaphragm) for at least a year, there have been two cases of endophthalmitis reported [19–22]. One of these occurred at an unspecified time to a patient with congenital aniridia [21]. The other was a case of endogenous endophthalmitis that occurred 4 months post-operatively because of long-term, systemic immunosuppressant therapy [19]. In one of the studies, 8 of 95 patients experienced acute hypopyon or anterior chamber fibrinous exudates, but all cases resolved within 3 months with topical therapy [20]. In another, 4 of 10 developed mild, acute uveitis which resolved with appropriate treatment [22]. A 2 year follow-up of 34 patients receiving a flexible iris prosthesis developed by HumanOptics and Koch found no cases of endophthalmitis [23].

The diagnosis of CPOE should always be suspected in cases of chronic, post-operative inflammation, but may be complicated by other potential etiologies such as uveitis due to retained cortical material or lens fragments, implant-induced chafing of iris remnant, sympathetic ophthalmia, or unrelated, systemic causes of chronic uveitis [7, 9]. In this case of severe, intraocular trauma followed by iris prosthesis implantation, all of these alternatives to CPOE, especially implant-induced inflammation, entered the differential and may have delayed diagnosis.

This case also reinforces the recurring nature of CPOE described in the literature [24]. For this patient, there were reductions in inflammation and pain in response to sub-Tenon’s capsule injections of triamcinolone and topical corticosteroid use which may have clouded diagnostic judgement in the short-term. However, as injections wore-off and topical corticosteroid treatment was tapered, inflammation and symptoms recurred and eventually led to explantation.

A limitation to this report is our assumption that intraocular, *P. acnes* infection was associated with iris prosthesis implantation. Though our assumption is based on the onset of signs and symptoms of intraocular inflammation after implantation similar to other reported cases CPOE, it is possible although less likely, that the infection originated in one of the two preceding intraocular surgeries, from removal of the protruding suture, or from contamination of the culture medium.

## Abbreviations

APOE – acute, post-operative endophthalmitis; CPOE – chronic, post-operative endophthalmitis; VA – visual acuity; OS – left eye
